# Medical and Physiological Commentaries

**Published:** 1841-04-01

**Authors:** 


### Medical and Physiological Commentaries. By *Martyn Paine*, M.D. A.M. 2 vols. 8vo. New York. London, 1840.

To sit down for the purpose of reviewing a work consisting of two closely printed octavo volumes, containing about sixteen hundred pages, requires no small degree of moral courage. But when the contents are devoted to the consideration of the most abstruse, metaphysical, pathological, and physiological subjects; every page abounding with references to, or quotations from, authors of every age, and of every country, the difficulty is greatly increased; and we approach the subject, if not with fear and trembling, at least rather as a task than as a pleasure. Such is the character of the work now before us: and it certainly is a favourable specimen of the laborious research and elaborate study of the writer. It reminds us of the German school of authorship: or of such books in our own language as Burton's Anatomy of Melancholy; and puts to shame those medical authors who favor the world with the results of their experience, observation, and acquirements, without indicating always the sources, whence they have derived their knowledge.

For our own parts, however, we candidly confess that we have a predilection for this latter class of writers: we are practical men, and we heartily subscribe to the maxim Dr. Paine appears to quote with approbation, "Ars medica tota observationibus."

We cannot, however, compliment him on adhering to his text, or carrying out the precept there laid down. His work, indeed, savours much more of the lamp than of the dissecting-room ; and of the study rather than the bedside of the sick. It will be a lasting monument of the author's profound and multifarious reading; and if a man have patience to wade through his pages, it will enable him to talk, and to dispute, on medical and physiological subjects; but we much question whether it will advance his knowledge as a practical man, or increase his perceptive powers in the detection and treatment of disease.

The opinion Lord Byron had of the Book to which we have above alluded as a literary work, coincides exactly with our own, respecting this as a medical one. "Burton's Anatomy of Melancholy," said he, "is the most useful book for a man who wishes to acquire the reputation of being well read with the least trouble. But among the medley of quotations the superficial reader must take care, or his intricacies will bewilder him. If, however, he has the patience to go through his volumes, he will be more improved for literary conversation than by the perusal of any twenty books with which I am acquainted: at least in the English language."

In attempting an analysis of Dr. Paine's work, it will be in vain for us, among the contrariety of opinions and doctrines brought forward, to give more than a summary of his own views: indeed to do this will often be no easy task. His style is so verbose, and so interlarded with quotations, that it becomes bewildering, and we are frequently at a loss to know his meaning. We shall endeavour, however, to lay before our readers such brief practical comments and reflections, as a careful perusal has left on our minds,· and by this means we trust we shall do good service to many, who, like ourselves, have but little leisure for the discussion of these recondite and speculative opinions. Indeed, we apprehend that elaborate works of this kind meet with but few readers; and we fear that the circulation of Dr. Paine's book will but ill repay the labour bestowed on its compilation. The taste of the age (in this country at least) is against them: besides, men in actual practice have no leisure for the perusal of abstract and controversial subjects, and the time of students may commonly be better employed, in anatomical and experimental researches, and in clinical observations. We do not make these observations from any disrespect to the author; or with any wish to detract from his great research, and peculiar merits; but they are in accordance with our own views, for we have been accustomed in our studies to follow the advice of that most accurate observer, Baglivi, whose writings are a perfect model both to imitate and to follow.

"In lectione librorum nunquam proficies, nisi prius in legendo methodum tibi comparaveris. Lectio librorum tumultuaria, inconsiderata & inexplebili quadam aviditate facta, mentem hebetat. Commoda, considerata & Doctorum virorum conversatione, atque experimentorum usu conjuncta, eandem fæcundat ac perficit. Et sicuti nimia ciborum ingurgitatio salubriorum valetudinem non affert, ita nec inexplebilis librorum lectio solidiorem doctrinam."*

The first section is devoted to the consideration of the vital powers. The author after criticising the definitions of Bichat, Lawrence, Müller, &c. &c.

---

* Baglivi, Prax. Med. Caput 7. De Præpostera librorum lectione.

propounds his own views, which, so far as we are able to collect them, are the following. Matter is endowed with certain "vital forces," distinct from its essence—which govern its phenomena, and which are susceptible of impressions from certain stimuli.

These stimuli are the remote causes of vital actions—whilst the "vital forces" in connexion with the organised matter, are the immediate source of the phenomena.

What is life? Life, philosophically considered, is a cause, and productive of results which constitute life in a popular sense. The functions are merely the results of the vital forces operating upon organised matter and called into action by certain stimuli. Life, therefore, virtually consists in the co-existence of these forces and a peculiar substratum. The soul exists in the fœtus; but like the senses is not displayed until certain external causes call its existence into activity. The resistance of the egg and seed to putrefaction, depends upon the presence of the "forces of life:" their activity, or growth, is produced by the peculiar stimuli acting on those forces. The elements of organisation are held in combination by the action of the vital forces, on the withdrawal of which they separate into their ultimate elements.

The conclusions he draws are—that life consists in the integrity of the vital forces associated with organised matter—that the vital actions are only the results of the foregoing conditions—that the vital properties are essentially distinct from organised matter itself, or any chemical or physical agency;—and that organic structure may exist entire without the properties of life—though the former is necessary to the existence of the latter.

In the second section the author treats with great severity the opinions of those philosophers who attempt to explain the phenomena of life on chemical or other analogous principles, and whom he terms the chemical physiologists. In a strain of irony, and in a spirit of unfair criticism, he reviews their writings, and by means of garbled and insulated extracts—by confounding them with their own conflicting opinions—and by not unfrequently misapprehending or mis-stating their views—he soon demolishes the doctrines (or, according to Dr. Paine, chimeras,) of such small fry as Prout, Philip, Davy, Bostock, Elliotson, Müller, et id genus omne. After thus laying prostrate names which we, in our simplicity, have almost held sacred in this department of science, we naturally expected to be enlightened by our author's own views, but, alas! we are left still in the dark.

We have the perpetual repetition of "vital forces," "vital powers," "properties," and such terms; but what do they explain, or to what practical purpose do they lead? They tend, in our opinion, to *mis*lead, and to mystify, rather than to enlighten; and will be just as useful to physiology, as the researches of Sir Isaac Newton would have been to astronomy, had his powerful mind been employed in discovering the causes of gravitation instead of investigating its laws.

No one, we apprehend, doubts the existence of "vital forces," "properties," or something inherent in living beings, totally distinct from chemistry, galvanism, or other analagous powers, and which can neither be produced nor imitated by any of these agencies. But in a secondary and practical point of view, there is no doubt, that many of the processes of life may be modified by chemical and mechanical causes—and consequently these sciences frequently lend their aid, not only in the explanation, but also in the treatment of various diseases and malformations incident to living beings.

The third section is occupied partly in defining the essence of life, and partly in criticising the opinions and definitions of others.

> "The essential principle of life," says our author, "is a simple substance, something analogous to, but distinct from, the soul itself, something intermediate betwixt spirit and matter, or in animals betwixt instinct and matter."

Assuming Scripture as a ground of argument, he says—

> "It is manifest that man was completed in his structure without life before he became endowed with a soul, and that the act which created his soul bestowed also the vital forces. One appears to be as much a new creation, distinct from the forces of dead matter as the other. When man was already perfected in his structure, he was without life. But by the act of breathing into his nostrils, his peculiar physical life and his soul were simultaneously created, and such is their companionship whilst life continues, that some philosophers have considered them identical. And how perfectly in harmony is all this with the exit of man. His soul and the vital forces leave the corporeal frame simultaneously: nor will either be restored but by another act of creative energy." 88.

He then triumphantly shews that—

> "The vital forces cannot be generated by matter, since upon them organisation depends: nor by the forces of physics, since these are perfectly incapable of restoring the structure, or even its elementary composition after the organised matter is decomposed, or of re-animating the machine after decomposition has begun." 92.

We need scarcely point out the incongruity of these passages: in the latter the author states that the vital forces are the *cause* of organization, whereas in the former it is shewn that man was *perfected* in his structure or organization *before* he was endowed with the vital forces: nor need we advert to the irreverent expression that "the soul will not be restored, but by another act of creative energy." We do not mean to insinuate that in this and similar passages, the author denies the immortality of the soul; but his language on this—the creation of man, and other sacred subjects, is so vague, and often so contradictory, that we fear doubts may be engendered in the minds of many of his readers.

Discrepancies of the kind to which we allude, may be found in almost every page of this Essay. For instance, in the passages we have quoted above, the author is compelled to have recourse to Divine agency for the creation of his vital forces; yet, in a note, he says that "the whole work of creation was miraculous, and therefore is not connected by any analogies with the subsequent processes of Nature;" and in page 10, he states—

> "Some able writers have lately appeared, who, admitting that life consists of a certain series of phenomena peculiar to organised matter, and having endeavoured to explode the entire doctrine which regards the forces upon which those phenomena have been supposed to depend—have proceeded so far as to affirm that the Deity himself is the immediate cause of all the phenomena of Nature.

The latter construction has arisen, in part, from the irresistible conviction that actions of all kinds require a certain power for their development. With this class of reasoners it will be difficult to argue, since their doctrine is a matter of faith and not of reason. There is no common ground between us."

Again, page 86, he accuses Müller of materialism, " in part from speculating on a subject so far beyond all human comprehension as the endowment of a fœtus with a rational, immaterial and immortal soul." Whilst, in page 13, he argues on the same point himself.

" Since, therefore, the fœtus, or a new-born infant, has as much a soul as man, we argue, that if the child sees, hears, tastes, smells and feels, as soon as it enters the world—the properties on which those functions depend, had a full existence in the fœtal state at the time of its birth."

The conclusions Dr. Paine appears to draw from his reasonings on these speculative points are—1st. That life in its essential principle is a simple substance. 2dly. That it is distinct from all physical, chemical, or other forces, and that its phenomena cannot be induced or explained by them. And, 3rdly, that there is a sort of tertium quid, a middle matter termed sympathy, which forms the connecting link between the vital forces and organised matter, and which receives external impressions and communicates them to these vital forces.

The appendix is devoted to an examination of the theories of electricity, galvanism, &c. as to their being analogous to nervous influence, or in any way explaining its phenomena; which the author denies in toto, considering the nervous influence to depend on a cause quite independent of them.

The next essay is on the philosophy of blood-letting, and extends over 300 closely printed pages. We will endeavour to give a summary of the author's views; but we confess, amidst the obscurity which pervades his essay, we can neither discover his meaning in many parts, nor his object in writing it at all. He professes to detect errors in almost every writer on the subject, and the theory which he propounds is something like the following.

The first effect of blood-letting is a contraction of the bloodvessels; this is brought about not from its diminishing the quantity of blood, but from its action on the vital forces. In leeching the contraction commences in the extreme vessels, and is propagated by sympathy—by continuity, or by remote consent, along the whole chain of vessels to the heart. The peculiar efficacy of leeching seems also to depend on some specific impression on the vires vitæ, which no other mode of abstracting blood can exactly accomplish: and one of the advantages is the effect kept up on the vital forces of the capillaries, and thence conveyed to the vascular system at large: hence, no matter how far the leeches are applied from the affected parts, the impression is propagated to it by continuity or remote consent.

This doctrine of the vital forces, and the contraction of vessels, explains, the author thinks, some anomalies in blood-letting:—for instance, less effect is produced, he says, by depletion in phrenitis than in pneumonia, because the sympathy between vessels of the same order is so great, that when those in a state of inflammation, refuse to contract the whole series throughout the body is maintained in a correspondent state. In phrenitis—

"The contraction of the cerebral vessels is partly prevented by the peculiarly modified state of their vital powers, and in part by the tendency of that modification to prevent a contraction of the corresponding vessels in other parts. The peculiarity of this modification arises from the nervous influence which is exercised upon the vessels of the brain in a state of inflammation; and is thus distinguished from the condition of the vital forces which affects the small vessels when the seat of inflammation is in other parts." 166.

Syncope, according to our author's doctrine, is a complex phenomenon. It is induced by the powerful influence of the vital forces of the capillaries, and the consequent universal contraction of the vessels; occasioning an influx of blood to the heart, and thus embarrassing its action. There is also an impression on the brain, which is produced by a diminished supply of blood, and by the sympathetic re-action.

"If, however, moral causes produce syncope, the primary affection of the brain consists only in some specific impression on its properties; and that its functions are only suspended by the failure of the heart's action. We infer also that the influence of the brain upon the heart, so far from being 'diminished,' is actually increased. This modification of the cerebral powers is constantly mistaken, as it appears to us, for a failure of functions." 176.

Blood-letting, then, operates invariably through the medium of the vital forces; and its phenomena cannot be explained on any mechanical or other principle. This is the fundamental doctrine of the author. It would be useless to follow him through his prolix reasonings and details—his endless criticisms and quotations from other authors—and his perpetual complaints that their practice is warped and influenced by their theories. "Mutato nomine de te fabula narratur."

His theory, as it appears to us, is mere assumption; unsupported either by experiments or facts. His reasonings, therefore, can be pertinently addressed to those only, who yield their assent to his assumption, nor will even they, we apprehend, generally admit his conclusions. But a controvertist trifles with his readers, when he grounds his arguments on the assumption of a theory which is denied by many, and doubted by more. Besides, to what practical purpose does this theory lead? it neither simplifies our knowledge of the powerful remedy it professes to explain, nor points out the cases proper for its adoption. It leaves, indeed, unanswered the very question with which he commences his enquiry. "How does blood-letting operate? How are diseased vessels unloaded, in some instances by the abstraction of small quantities of blood; when in other cases, under apparently the same circumstances, a great extent only of the remedy will effect the same results?" P. 121. Indeed nothing can shew the inconclusiveness as well as uselessness of his researches so clearly as his own admission.

"As to the assumption," says he, "of any particular symptom or circumstance as a rule for blood-letting, we hold it to be indefensible, and shall endeavour to prove it so in subsequent sections of this essay." P. 237.

We shall, however, best enable our readers to form their own opinion of the style and value of Dr. Paine's comments on other authors, and of his own practical information, by selecting a few of his criticisms on some of the more recent writers on blood-letting.

Dr. Wardrop says, "The leading symptom by which the constitutional disturbance demanding venesection is indicated, will be found in the quality of the pulse."

Our author states, "There is scarcely any symptom, per se, that is less to be trusted to than the pulse, unless it possess certain positive characters." P. 233.

Again, page 237, "We think that very few will agree with Dr. Wardrop that 'there is usually no appearance of the buffy coat in blood removed from persons affected with violent inflammations, until the latter stage of the disease, and at the very period when the further abstraction of blood would be pernicious.' "*

"On the contrary, indeed," Dr. Paine says, "we find in 99 of 100 such cases, that the buffy coat is presented at the *first* bleeding and has disappeared more or less when the further abstraction of blood would be pernicious."

"Again," Dr. Wardrop says, "in almost every case where venesection is necessary, there is present along with the disturbed action of the arterial system some local pain more or less severe." "Now this," says our author, "is notoriously not the case in very many instances of venous congestion, in many chronic inflammations, and often in severe cases of pneumonia; in all of which blood-letting may be indispensable." P. 237.

Dr. Arnott states "that it is a great modern improvement in the practice of the healing art in bleeding for the cure of inflammation, to take away the blood *as quickly as possible;* since intense inflammations of the brain, lungs, bowels, &c. are equally removed by faintness, whether it happens after the loss of ten ounces of blood, or of fifty." "This," says our author, "is a fallacy."

This quotation from Dr. Arnott is from his Elements of Physics, p. 470. Dr. Paine adds, in a significant note—"Hence too, appears the fallacy of applying the 'Elements of Physics' to considerations of this nature.

In the same ex cathedra style Dr. Paine criticises the opinions of other writers. He thus concludes his strictures on Dr. Marshall Hall. "But the *most exceptionable* part of Dr. Hall's rules, as it appears to us, applies to the repetition of blood-letting." "If much blood has flowed," says Dr. Hall, "before incipient syncope has been induced, re-visit your patient *soon* and you will probably have to repeat the blood-letting, in consequence of the severity of the disease; especially if you were not called in early in the first instance. If, on the contrary, *little* blood has flowed, *neither does the disease require*, nor would the patient *bear*, farther general depletion."

We have made these quotations, which we might extend to any length, partly to exhibit the style of Dr. Paine's criticisms, and partly to give greater currency to the valuable suggestions which he so strongly reprobates. As we write for practical men, we would appeal to them whether the opinions laid down in the quotations from the three eminent writers above-named, are not consonant with their own experience. For our own parts, we consider these and similar passages interspersed through the writings of such men as Hall, Wardrop, and, we may add, of Sydenham, Heberden, &c. to constitute their peculiar interest and value. It is by recording observations

* On Bloodletting, p. 41.

made at the bedside of the sick, and verifying them by experience, that the science of practical medicine is to be advanced; and not, in our humble judgment, by framing theories and then making our practice subservient to them. Facts and observations are our landmarks: for diseases and their symptoms remain the same, although the theories which profess to account for them change with almost every writer. We do not now read Cullen and Darwin to know their theories—but to know their facts and their practical inferences. Dr. Paine's theory may or may not be true, bleeding may operate *only* " by producing its direct effects upon the vires vitæ of the capillaries by modifying their action;" or it may operate in some other way; but we wish to know in what cases of disease bleeding is proper: and if proper, in what way blood should be abstracted. This our author's theory does not teach. It can only, indeed, we apprehend, be acquired by our own observation, aided by the recorded experience of others. He is consequently the best practitioner who is the most accurate observer, and who has sagacity to profit by and to arrange recorded facts. Had we, indeed, a theory founded upon nature, that should bind together the scattered facts of medical knowledge—and converge, as it were, into one point of view the laws of life, it would contribute much to the interests of medical science. But theories framed to explain the causes and nature of disease, and the modus operandi of medical agents, have advanced, and probably will advance, but little the practice of medicine. They may assist us to explain some physiological facts, or the phenomena of some diseases; but the successful treatment of them must mainly be learnt in the school of experience.

Dr. Paine's experience may have been extensive; but the perusal of his work leads us to the conclusion that his practice is founded on theory rather than on observation. In every section of his essay, while he displays both industry of research and dexterity of applying quotations and references; he has at the same time, through eagerness to establish his favorite position, called into his service a number of remarks, many of which are greatly overstrained in their application, and others if strictly examined will be found to disserve his cause. Through the same anxiety to follow up his theoretic views, he advances opinions in various parts of his essay which if put in practice would, we think, prove highly dangerous. In page 337, he says, " We hold it (bloodletting) to be more important in infancy, under equal circumstances, than at any other age; and this ratio increases as we ascend to the hour of birth." In page 361, among the aphorisms with which he concludes his essay is the following.

" Blood-letting is equally safe at all periods of life, but is *most indispensable* in old age."

Now, we submit, that if Dr. Paine's practice had been guided by experience instead of theory, he would not have come to conclusions, and have made unqualified assertions so fraught with danger.

We do not deny that cases may occur in extreme infancy as well as in extreme old age which require bleeding; but these instances are very rare, and when they do arise, the greatest caution must be observed.

The same recklessness in recommending blood-letting pervades the work. In all the modifications of scrofula, tubercular consumption, erysipelas, &c. bleeding is the sheet-anchor; and although the author, having no guide but his theory, is occasionally at a loss whether he shall bleed or not; yet instead of giving the patient the benefit of the doubt—he says, page 238, "If, after surveying the whole aspect of a case, we remain in doubt about the propriety of abstracting blood, we generally take out our lancet and bleed the patient." He then adds, "We every where see victim after victim sacrificed to timid admonitions and worse example; whilst you and all of us know that it is a rare phenomenon that a patient is slain, seldom injured by the lancet."

We are apprehensive that these phenomena will henceforth become much less rare if the less "timid admonitions" of this work be implicitly followed.

The next and concluding essay of the volume is on the humoral pathology, and extends over nearly 400 pages. It consists almost entirely of criticisms, quotations and references.

The author, in pursuance of his own views, regards the phenomena of disease, its causes and treatment, as resulting from his doctrine of the vital forces: hence he exposes, and attempts to controvert the opinions of all writers, from Galen downwards, who advocate the humoral pathology.

It would be vain to attempt, and useless were it possible, to give a regular analysis of this elaborate essay. The endless criticisms of the author elicit no useful information; and he not unfrequently perverts the opinions of writers, and even distorts their facts, if they militate against his preconceived views. On the other hand, he arrays on his side of the question as solidists or vitalists, men whose writings cannot be so fairly interpreted; and many living writers, we apprehend, will be surprised to find themselves in such company.

For instance, he designates this Journal as "that stable solidist The Medico-Chirurgical Review;" and adds, "we consider Dr. Johnson himself in all respects a solidist." Now we need only appeal to our readers, whether this Journal has not invariably advocated the opposite doctrine, indeed we could refer to fifty passages in proof of our assertion were it necessary. Ex uno disce omnes.

The question at issue, as the author fairly states it, is this—"Whether foreign morbific causes and remedial agents, in their ordinary modes of operation, produce their primary effects upon the solids or upon the blood, and the latter become the cause of disease in the former; whether we have hereditary humors, as gout, scrofula, &c. &c." We are satisfied with the answer of M. Andral to this question. "Physiology," says he, "leads us to the conclusion, that every alteration of the solids must be succeeded by an alteration of the blood: just as every modification of the blood must be succeeded by a modification of the solids. Viewed in this light, there is no longer any meaning in the disputes between the solidists and the humoralists."

This view of the matter, however, does not satisfy Dr. Paine. He is an exclusive; and will have no modified or half-and-half doctrines. He is a confirmed solidist; and demolishes with unsparing criticism hosts of authors whom he quotes, whose views militate against his own.

The second volume commences with the "Philosophy of Animal Heat." The author reviews the opinions of those writers who explain the phenomena of animal heat on chemical principles. He shews clearly, we think, that there are many anomalies that cannot be accounted for on the theory that animal heat results from respiration, and the changes induced on the blood by that process. His own views on this subject may be deduced from the outline of his doctrine of the vital forces we have above attempted to explain.

" The power of generating animal heat relates to the vital forces and to nothing else." Heat is secreted or eliminated through the united agencies of these powers from the blood, in the same manner as the bile or gastric juice. This is certainly a very plain and simple statement of the matter. But there is no great novelty in it. Hunter maintained the same opinion.

" It is most probable," says he, " that the power of generating heat in animals, arises from a principle so connected with life, that it can and does act independently of circulation, sensation and volition; and is that power which preserves and regulates the internal machine." * We apprehend that writers subsequent to Hunter, who ingeniously attempt to explain the phenomena of animal heat on chemical principles, admit all that he or our author does on the necessity of a primum mobile or vital force. But as this vital principle usually makes use of chemical or mechanical agents for it purposes, we may, by investigating the laws of these agencies, be assisted in explaining some of the phenomena of animal heat, and in modifying or controlling its force when unduly or morbidly excited or diminished.

It will be unnecessary to detain our readers with any extended remarks on the subsequent essays of this volume. The same views and reasonings are made use of in all. In the next, " On the Philosophy of Digestion," the author states, that " the gastric juice is a substance, sui generis, endowed with vital powers—that it can only be generated by a living stomach —that it cannot be imitated by art; and that through its agency alone digestion is performed." These are truisms which few, in the present state of our knowledge, will be hardy enough to controvert; yet, as in the case of animal heat, some will be disposed to think that the function of digestion may be improved or impaired by chemical agencies; which, if we understand Dr. Paine, he utterly denies.†

According to him, medical philosophy, and its practical application, have nothing to hope for from chemistry.

The appendix to this essay contains some sensible and well written remarks in opposition to the theory of spontaneous generation.

The succeeding essay is a review of the various theories of inflammation. All are rejected by our author; and his own, the vital theory, considered the only true one.

The next essay is on " The Philosophy of Venous Congestion." It is most elaborate; occupying more than half the volume. As it consists, however, mainly of quotations and criticisms, we shall not attempt to analyse or abridge it.

Two essays on the " Comparative Merits of the Hippocratic and Anato-

---

* Hunter's Observations on Certain Parts of the Animal Economy, p. 91.

† For some interesting experiments on "Artificial Digestion," we beg to refer our readers to two essays by Professor Müller and Dr. Schwann, in the " Archiv. fur Anatomie und Physiologie," for 1836.

mical Schools," and "On the Principal Writings of P. Ch. A. Louis, M.D." conclude the work.

In the brief sketch we have given of this very voluminous and erudite performance, we have endeavoured to lay before our readers the peculiar views and opinions of the author. Although we dissent from many of his conclusions—regret the paucity of his facts, and have been bewildered by his reasonings and arguments; (so much so as perhaps to lay ourselves under the imputation—" damnant quod non intelligunt,") yet we willingly award to him the merit of multifarious reading and research; and of untiring zeal in the support of his doctrines.

